# Lecture on Delirium Tremens—Phthisis Pulmonalis—Meningitis—Pathological Anatomy of Gangrene of the Lungs and Meningitis

**Published:** 1839-12-21

**Authors:** W. W. Gerhard


### CLINICAL LECTURES.

### PHILADELPHIA HOSPITAL.

*Wednesday, December* 11, 1839.

### LECTURE ON DELIRIUM TREMENS—PHTHISIS PULMONALIS—MENINGITIS—PATHOLOGICAL ANATOMY OF GANGRENE OF THE LUNGS AND MENINGITIS.

BY W. W. GERHARD, M.D.

*No.* 5—*Winter Course.*

I BRING before you to-day the patient whom you saw on Saturday last, suffering with well-marked symptoms of *delirium tremens*, for the purpose of showing you the successful termination of the case. He has perfectly recovered from the attack, with the exception of slight giddiness, a symptom which is generally the last to disappear. The contraction of the pupils is no longer to be observed; the countenance has resumed its natural appearance; the intellect is free from disturbance; and the tremours of the limbs have nearly ceased. This disease is generally very rapid in its course, terminating in recovery or death in a very few days. Chronic cases are exceedingly rare.

The patient has had two attacks before the present one, and came to this hospital on both occasions: both attacks were attended by convulsions. The treatment has consisted in the use of brandy, porter, assafœtida, and other stimulants; opium was not administered. On Saturday last, (the day of his entrance,) the following prescription was ordered:

℞. Ammoniæ Carb.

Camphoræ āā ℈j.

Pulv. Capsic. ℈ss.

Lac Assafœtidæ ℥vi. M.

S. A tablespoonful to be taken every hour. During the night of the same day he had convulsions, and was unable to sleep; brandy was then given him. On the morning of the 8th he was much better; the convulsions had subsided; pulse ninety-six, and soft; skin moderately warm. During the day, however, he was much agitated; wished to kill himself, and begged that he might be killed quickly. Brandy was again given him, by which he was much relieved; he took thirty-six ounces in all: he has continued to improve ever since. Last night he slept soundly, and this morning, as you see, is quite well. The method of treatment which I have pointed out, viz. the more frequent administration of brandy and other stimulants in the severe cases of delirium tremens, with some other modifications in the treatment, has considerably diminished the mortality of delirium tremens in this hospital, as I shall take some future occasion to prove to you.

### PHTHISIS.

I shall now continue the subject of pectoral diseases, and present you another case of phthisis pulmonalis, with remarks, which may serve to give you some further knowledge of its history and diagnosis. The case before us is another example of the ordinary variety of phthisis, which commences gradually, and without any assignable cause. The patient is a printer, forty-seven years of age, formerly of intemperate habits, but since the commencement of the disease has been perfectly temperate. Last winter a cough commenced, but so gradually that the patient is unable to fix the time of its first occurrence; during the last four months it has been much more severe and constant, and accompanied by hoarseness. There has been at no time spitting of blood; the expectoration was at first mucous, afterwards muco-purulent and somewhat nummular: this change in the character of the expectoration took place about the time that the hoarseness commenced. The left side of the chest is somewhat contracted; the respiration is cavernous, with distinct pectoriloquy. These signs show the existence of a cavity, but it seems to be rather of an indolent nature. The hoarseness in this case is owing to inflammation attacking the larynx, and causing thickening of its lining membrane, and of the vocal cords: this inflammation often passes into ulceration; it is sometimes painful, but often there is scarcely a slight tickling felt at the part. I find on making firm pressure upon the larynx, that the patient complains of no pain, except just below the thyroid cartilage. This inflammation is the result of the irritation of the tubercular matter and other discharges which pass over the bronchial membrane, and it may take place at several different points of the respiratory passages—the larynx, the trachea, or the bronchi. This variety of inflammation is strictly secondary, and very different from that which occurs at the early stages of phthisis, in which the tracheal irritation is the cause developing tubercles, and not the result of their softening. There is generally considerable soreness attending the inflammation when the phthisis is acute; in more chronic cases it is slight, or altogether absent. This inflammation of the air-passages, in the cases of which I speak, is secondary; in others, as I have already repeatedly remarked, it may precede the development of phthisis. The other symptoms in the present case are of the usual kind; the skin is dry and harsh; there is general emaciation, with roundness and prominence of the ends of the fingers; the pulse is quick, tense, and irritated; there are chills about the middle of the day, followed by fever in the afternoon, and cold sweats at night. The fever, therefore, has the regular paroxysmal character of hectic.

You have now seen cases of several varieties of phthisis, differing in their origin and progress: it is time to say something of the diagnosis of this affection. The diseases with which it is most liable to be confounded, are bronchitis, pneumonia, and pleurisy, whether of the acute or the chronic form; since phthisis, also, may be either acute or chronic. It may likewise be accompanied by any of these inflammations, and it becomes important to distinguish such cases from those of simple inflammation. The diagnosis of tubercular disease depends both on the general and the local signs. The first circumstance to be attended to, is the *general appearance* of the patient. The frame is emaciated; the skin is of a pale and earthy aspect; there is a restless expression of countenance, indicating the workings of a slow disease, and entirely different from the alteration of the features which attends acute disorders. The emaciation shows itself very early in the hands and fingers,—the ends of the latter retaining their fulness for some time, and appearing rounded and prominent; the nails are likewise turned inwards. Emaciation is a sign of great importance in the diagnosis of phthisis and other tuberculous diseases; particularly if you find that the patient is losing flesh, although he eats nearly as much as in good health. When this sign is conjoined with others indicative of phthisis, the diagnosis becomes almost certain. The earthy hue of the skin is attended by a bluish tinge of the sclerotica, which very often occurs in phthisis.

We likewise observe a change in the *circulation*. There is a peculiar fever attending tubercular disease, and characterized by a quick, jerking pulse, the result of excessive irritation. This fever is accompanied by chills and sweating,—the former being at first very slight, while the latter is much more copious than in most other acute diseases. This peculiar condition of the pulse and general febrile excitement, are most important in the diagnosis of general tuberculous disease of an acute kind. It is, however, quite as well marked when the tubercles are rapidly developed in the lungs, as when they are deposited at the same time in several of the organs. The observation of these general signs should cause us to suspect the existence of phthisis, and lead us to the examination of the local signs. If, upon an inquiry into the latter, we are unable to ascertain the existence of any other disease, we are led, by a process of exclusion, to a confirmation of our original suspicions.

We also derive some important points of information from a consideration of the *predisposing causes* of the disease. Thus, the *sex* exercises some influence; phthisis is rather more frequent in females. *Age* is a more important circumstance: phthisis is most frequent in young persons, and is rarely met with after the age of thirty-five. When it does occur after this period, it generally depends upon some accidental cause, as inflammation, &c. The *course of the disease* is another point to be considered; phthisis is in most cases slow in its progress: the diseases with which it is liable to be confounded are more generally acute.

The last and most important circumstance in the diagnosis of this disease, is an attention to the *local signs*. The first of these is usually an uneasy sensation in the chest, very different from the pain of inflammation. It varies greatly as to its position, sometimes shifting from one side to the other, or felt under the sternum. Pain, however, may be present in the commencement of phthisis, when it is complicated with intercurrent pleurisy. The cough is constant, but rather more severe at night, or early in the morning, than throughout the day; it is at first short, and so insignificant as hardly to attract notice, and attended by mucous expectoration; in the progress of the disease it becomes loose, and the expectoration is muco-purulent and nummular; in a still more advanced stage, the cough is very loose,—the expectoration consists of pus mixed with softened tubercular matter, and loses its nummular character.

The *physical signs* are not obvious at the commencement. The first thing observed is generally a feebleness of respiration at the upper part of the lungs, which afterwards changes into rudeness; this arises from the obstruction of the lung by the tuberculous deposit. We next perceive a crackling sound under the clavicle, indicative of the commencement of softening. There is likewise a dulness on percussion at the summit of the lung. In the advanced stages the respiration is gurgling, cavernous, and amphoric; respiration with pectoriloquy.

There are certain *secondary symptoms* regularly occurring in phthisis. One of these is hectic fever; at first the fever attending tuberculous disease is not hectic; I have pointed out the difference in a previous lecture. When hectic is developed, it is recognised by the chills and sweating which accompany it; the flush on the cheeks, &c.; it is always paroxysmal. The loss of appetite, and decline of digestive power, do not depend upon the deposite of tubercles, but upon the fever which attends it; they differ in no way from the same symptoms which ordinarily accompany febrile diseases. The diarrhœa of phthisis, however, does depend upon the formation of tubercles. It is intermittent, very irregular in its character, occurring sometimes frequently in the course of the disease, sometimes only once or twice. Its immediate cause, in most cases, is the development of tubercles in the glands of Peyer, which, consequently, become inflamed and ulcerated, but diarrhœa in phthisis may arise from the same causes as in other diseases, in the latter case, its symptoms and progress are entirely similar. Hæmorrhage is another of the accidental or secondary symptoms of phthisis; the blood is either discharged directly from the lungs by a slight cough, or it is swallowed, and afterwards ejected from the stomach. If the hæmorrhage be profuse, that is, not less than two or three ounces in a day, it is considered almost pathognomonic of phthisis, especially in males; in females, it is not so certain as a diagnostic sign, for hæmorrhages from different parts of the body often arise from suppression of the menses, &c., and are, in fact, vicarious discharges; but in men, as I observed in the last lecture, in at least five cases out of six, abundant hæmoptysis arises from tubercular disease of the lungs. The fact that hæmoptysis is a very important sign of phthisis, has been long known, but Dr. Louis has rendered the profession a decided service, by proving that the value of the sign was even greater than had hitherto been believed. Many cases of this hæmorrhagic variety of phthisis terminate in recovery; it is, in fact, the least unfavourable form of the disease, and therefore the value of the symptom is sometimes underrated, because to the minds of many physicians, the words phthisis and death are considered as almost inseparable. This form of the disease is probably less fatal than others, simply because the flow of blood relieves, to some extent, the vessels of the lungs, and appears to be a natural safety-valve, which diminishes the tendency to the tuberculous secretion.

### MENINGITIS.

The patient, who is now before you, a man about forty years of age, was presented, on Saturday, as an instance of phthisis, accompanied by bronchitis. I now bring him forward to illustrate another disease,—meningitis. This disease has been sometimes observed in the French hospitals to assume an epidemic character; and this is the case at present in our own hospital. In the last few days, eight or ten cases of the disease have occurred. This patient was seized on Sunday with delirium, preceded by flushing of the face; the delirium has continued, with intermissions, up to the present time; it generally subsides in the morning, and is most severe at night; it is, for the most part, first observed at night in meningitis. This morning, the patient is weak, but suffers no pain in the head; the mind is clear, and the violence of the disease in every respect much abated.

*Case 2d.*—This case is somewhat similar to the preceding. The patient entered the hospital on the 31st of November. He is a labourer; has enjoyed good health until the 4th of August, with the exception of an attack of intermittent fever some years since, and palpitation of the heart following it. In August he was taken with chills and fever, for which he was treated in the Pennsylvania Hospital; he was cured for a time, but relapsed. On Sunday, cerebral symptoms were developed; he was much agitated during the day, and became delirious at night. On Monday, he complained of pain in the region of the transverse colon, and over the liver; this was relieved by a blister, and injections of mucilage, with a small portion of laudanum. Yesterday the signs of cerebral disorder were more marked; cupping and cld applications to the head, were then employed. To-day there is some improvement. The pain in the upper part of the abdomen, I suppose to depend on inflammation of the serous coverings, involving the serous membranes above and below it. This is proved by the enlargement, tension, and pain on pressure, in the right hypochondrium; and by the pain which is felt on percussing between the ribs. Inflammation on one side of the diaphragm frequently passes across it, and affects the parts on the opposite side; thus hepatitis may give rise to pleurisy; or inflammation in the chest may involve the liver, as occurs in pneumonia biliosa. Along with the serous inflammations already noticed, the arachnoid membrane in this case is also affected; in which circumstance we have an illustration of the tendency which serous membranes have to become similarly affected in different parts of the body at the same time. This inflammation of the arachnoid is properly a serous inflammation, though it does not generally affect the free surface of the membrane, (as is usually the case in other inflammations of this character,) but the surface which is adherent to the pia mater.

*Symptoms of meningitis.*—There is first an obscure sense of uneasiness in the head, which may be readily confounded with that arising from various other causes; then there is dizziness and pain, affecting either the fore part of the head, or the whole, according to the seat of the inflammation; this is most commonly when the inflammation is most severe at the base of the brain; the pain is apt to extend nearly over the whole head. We also observe a change of countenance in the patient before us; there are occasional and transient frowns, with deep vertical wrinkles at the root of the nose; a severe expression of the features, or a wild staring look. The eyes should be closely attended to: they are very bright, but not injected; the pupils are contracted, owing to greatly increased sensibility to light: in children, these signs are especially important. There is a contraction about the mouth, and constant involuntary motions of the lips; these motions incline rather to the left side of the body, but there is not yet any thing like a fixed distortion of the mouth. The inclination which the lips have to the left side, is owing to the inflammation being more severe in the right side of the brain than the left. Another consequence of this is, a slight rigidity of the left arm. To ascertain the existence of rigidity in the arms, I raise them into a vertical position, and extend the forearm,—then letting the latter fall, while I hold the arm fast, I can judge from the relative freedom of the motion, whether any rigidity exists; and in this way, also, you should compare the two arms together. Rigidity of the upper extremities is usually accompanied with a slight flexion of the hand. Of course, rigidity of the lower extremities cannot be ascertained in this way, and the disease is generally considerably advanced before its existence becomes manifest; it is very rarely accompanied with contraction of the flexors. All these symptoms of arachnitis and the other inflammations, are entirely local; the pulse is not much affected, nor has jaundice yet resulted from the inflammation of the liver, though in all probability it will occur before long. At this moment I observe a symptom which has not occurred before in the present case—strabismus; this is another frequent symptom of meningitis; and, like those just mentioned, is owing to the cortical substance of the brain participating in the inflammation of the membranes. The eye is the best standard by which to judge of the extent to which the senses are affected; for the other senses are not so subject to the observation of the physician,—and it is impossible in most cases to derive from the patient any accurate information as to their condition, because, from the nature of the disease, he himself is incapable of perceiving any irregularity in the actions of the system. Delirium is generally preceded by agitation of mind, which lasts through the day, and at night passes into delirium. As the signs of meningitis depend upon the brain, the diagnosis is, in most cases, easy, unless the disease should come on very slowly, or secondarily to some other affection, as in typhus or typhoid fever, where delirium often arises from a change in the condition of the blood, or in inflammation of other organs, in all of which cases true meningitis may occur as a secondary lesion. The local signs are more particularly worthy of attention in affections of the brain and its membranes, because of the great importance of the organ, and because, in many cases, there are very few geneial symptoms which can lead us to a correct conclusion; we have, therefore, to attend to the most trifling local symptoms which indicate the beginning of organic disorder of the brain. I omit for the present the general symptoms.

When meningitis is chronic, all the preceding signs appear more slowly, and paralysis occurs sooner than in the acute form. Paralysis, in these cases, first affects the mouth and tongue, producing a thickness of voice,—then the lower extremities, as is shown by the halting gait with which the patient walks. For some time the change in the intellect is very slight in chronic meningitis; but there is constant dizziness and pain, which should cause us to suspect the existence of meningitis, even before the occurrence of paralysis.

*Treatment of acute meningitis.*—The first measure ought to be a bleeding from the arm to such an extent as to produce a decided impression. But general bleeding is only proper in the early stages of the disease; we must afterwards rely upon local bleeding, which may be repeated again and again; as cups are much more convenient than leeches, you will generally prefer them. Pounded ice constantly applied to the head by means of a bladder, is also a valuable remedy; but its effects must be watched, and if it produce paleness of the face and chilliness, it ought to be removed. Purgatives are also very necessary, and of this class of remedies mercurials appear to be the best; it is sometimes beneficial to push them even to ptyalism. Entire abstinence from all exertion, mental or corporeal, must be rigidly enforced. A more detailed account of the treatment, the time allowed to this lecture does not allow me to give you.

At the last lecture I showed you a patient suffering with inflammation of the pleura, and the membranes of the heart. Since that time the pulse has continued quick and irritated, like that of phthisis; but the rasping sound still continues during the systole. The case has been treated by cupping, blistering, and digitalis combined with calomel. In these cases of pericarditis and endocarditis, the diagnosis depends partly on the general, partly on the local signs. The latter consist of the pain in the præcordial region, the alteration in the action of the heart, and the sounds yielded by percussion and auscultation. The general symptoms, as the state of the pulse, the œdema, &c., are much less to be relied on. The patient complains of no tenderness in the abdomen; very slight pain in the præcordia; indeed, his complaint is of dyspnœa and cough, instead of pain. The pulsation of the heart is disordered, and the rasping sound is quite distinct. This rasping sound resembles the bellows sound, but is distinguished by its greater harshness. They are both heard in this patient, and indeed in most cases, during the systole or first sound of the heart, and arise from the want of a sufficient outlet of the blood through the pulmonary valves of the aorta, or from regurgitation of the blood through the mitral valve, when any obstacle prevents this from closing completely during the systole of the heart. The contraction of the orifice of the aorta is by far more frequent in these cases of endocarditis than the dilatation of the mitral valve, and we therefore suspect the occurrence of the former lesion as an attendant upon the inflammation. There is another cause of these morbid sounds, that is, the irritation of the inflamed heart, causing it to contract spasmodically and too rapidly on the passage of the blood.

The case of gangrene of the lungs which I showed you last week has since terminated fatally, and I will now present to you the results of the post mortem examination. You will recollect that I then stated that the disease affected the lower lobe of the right lung: it rapidly extended itself, and the patient sank in proportion. There is, in fact, no specific treatment by which the disease can be arrested; all that we can do is to support the system until nature accomplishes the cure, if such is her design. The pathological appearances of gangrene of the lungs are closely connected with, and explain, the symptoms during life, viz., fœtor of the breath and expectoration, lividity of the countenance, and the physical signs of a cavity in the chest. This case occurred in consequence of the patient falling into the river: the gangrene probably commenced about two weeks afterwards, and had continued for several weeks previously to his entrance into the hospital: since that time it has been constantly advancing. If the progress of the disorganization could have been checked, it is probable that the case would have terminated favourably, for the mischief already done was not necessarily fatal. You will at once perceive the excessive fœtor of the lung, and its dark green colour over the lower lobe. As I lift it up the surface of the lung sinks towards the cavity, which occupies the greater part of the lower lobe of the left lung. The cavity rapidly increased in size during the last days of life, as was proved by the enormous quantity of matter expectorated, amounting to at least a pint in the course of the twenty-four hours. The immediate cause of death, however, was dyspnœa, arising from inflammation attacking the heart.

Pleurisy, in a greater or less degree, always attends gangrene of the lungs; here we have the proof of its existence, in the false membrane which covers the surface of the pleura. This inflammation of the pleura, producing false membranes, and adhesions of the lung to the ribs, tends to prevent the perforation of the pleura, and the discharge of the gangrenous matter into the cavity of the chest. From the existence of amphoric respiration, perforation might have been suspected in the present instance, had we seen the patient only on the last day or two of life: but you now see that no such thing has occurred, and that the amphoric respiration was owing to the great size of the cavity. The lung is much softened around the cavity, and yields readily to the knife. The cavity is large enough to hold the fist: it is seated entirely in the lower lobe, having been first formed at its upper part: the disease very rarely attacks the upper lobe. By an examination of the walls of the cavity, we will be able to determine whether any process had commenced for the cure of the disease: when this does take place, it is by means of a false membrane which is formed around the cavity, and secretes pus and mucus, as is shown by the character of the sputa. But here we see no appearance of a false membrane, nor of pus or mucus: the cavity contains a gangrenous slough, and a quantity of the offensive matter which was so copiously expectorated during life. The walls are blackish, and gradually pass into the healthy lung. The existence of cavities formed in this way, is known by nearly the same physical signs as those which are observed in phthisis; but the fetor of the breath and expectoration is sufficient to distinguish the two diseases, independently of various other circumstances. The mucous membrane of the bronchi is inflamed in consequence of the passage of the gangrenous sputa over it: in some cases this matter is swallowed, and produces severe diarrhœa. The upper lobe of the left lung is healthy, with the exception of a few miliary tubercles scattered through its summit.

The right lung became inflamed in the progress of the case. The pleura is covered by a false membrane of the consistence of cellular tissue, which is very brightly injected. This inflammation was one of the causes of death. The substance of the lung is healthy, with the exception of a slight engorgement and induration in the centre, which probably constitute the first stage of gangrene, and a few tubercles in the upper lobe.

Upon examining the heart, we find traces of former pericarditis, in the patches of lymph on the surface of the pericardium, and the serous effusion into its cavity. The heart is of the natural size: its muscular structure is in the normal condition, and its lining membrane is also nearly natural, but not entirely so; there is a slight thickening in the left ventricle, and also of the semilunar valve of the aorta. The right side of the heart is frequently found perfectly healthy, though the left be greatly diseased: in this case we perceive merely a slight opacity of the internal membrane of the right ventricle. The valves of the pulmonary artery are quite healthy.

The spleen is enlarged and softened; this appears to depend upon the vitiation of the blood produced by gangrene, in the same way with the livid hue of the skin. The liver is fatty, and of a lighter colour than natural. This fatty degeneration in males is frequently the consequence of intemperate habits: it is also very common in phthisis, more particularly that of females. In the progress of this alteration, the cellular tissue uniting the acini becomes more developed than natural, while the acini seem to disappear, their places being occupied by fat.

I next show the brain of a subject who died of meningitis. The disease in this case was the result of suppuration of the ear, a cause more frequently operating in children than in adults. When the brain was taken from the subject, the meninges were injected with blood, which has now nearly disappeared: we observed, however, an evident thickening, and a deposition of a puriform substance, extending from the chiasm of the optic nerves, along the fissure of Sylvius, on the left side of the brain. We likewise observe opaque spots in the membranes elsewhere, similar to those just seen in the pericardium.

Here is another specimen of the same disease. The meningitis in this case occurred in a lunatic. The thickening of the membranes is a proof that the inflammation was chronic, while the bright injection shows that it afterwards became acute, as frequently happens in every kind of inflammation. The ventricles contain the natural quantity of fluid; the substance of the brain is firm, and of the natural appearance. The disease, therefore, was confined to the meninges.

